# Preparation of Solid Self-Nanoemulsifying Drug Delivery Systems (S-SNEDDS) by Co-Extrusion of Liquid SNEDDS and Polymeric Carriers—A New and Promising Formulation Approach to Improve the Solubility of Poorly Water-Soluble Drugs

**DOI:** 10.3390/ph15091135

**Published:** 2022-09-11

**Authors:** Fabian-Pascal Schmied, Alexander Bernhardt, Sandra Klein

**Affiliations:** 1 Institute of Biopharmaceutics and Pharmaceutical Technology, Department of Pharmacy, University of Greifswald, Felix-Hausdorff-Straße 3, 17489 Greifswald, Germany or; 2 Research, Development & Innovation, Evonik Operations GmbH, Kirschenallee, 64293 Darmstadt, Germany

**Keywords:** copolymer, amorphous formulations, poorly soluble drug, drug release, hot melt extrusion, nanoemulsion, celecoxib, fenofibrate, efavirenz

## Abstract

The present study focused on a new formulation approach to improving the solubility of drugs with poor aqueous solubility. A hot melt extrusion (HME) process was applied to prepare drug-loaded solid self-nanoemulsifying drug delivery systems (S-SNEDDS) by co-extrusion of liquid SNEDDS (L-SNEDDS) and different polymeric carriers. Experiments were performed with L-SNEDDS formulations containing celecoxib, efavirenz or fenofibrate as model drugs. A major objective was to identify a polymeric carrier and process parameters that would enable the preparation of stable S-SNEDDS without impairing the release behavior and storage stability of the L-SNEDDS used and, if possible, even improving them further. In addition to commercially available (co)polymers already used in the field of HME, a particular focus was on the evaluation of different variants of a recently developed aminomethacrylate-based copolymer (ModE) that differed in M_w_. Immediately after preparation, the L-SNEDDS and S-SNEDDS formulations were tested for amorphicity by differential scanning calorimetry. Furthermore, solubility and dissolution tests were performed. In addition, the storage stability was investigated at 30 °C/65% RH over a period of three and six months, respectively. In all cases, amorphous formulations were obtained and, especially for the model drug celecoxib, S-SNEDDS were developed that maintained the rapid and complete drug release of the underlying L-SNEDDS even over an extended storage period. Overall, the data obtained in this study suggest that the presented S-SNEDDS approach is very promising, provided that drug-loaded L-SNEDDS are co-processed with a suitable polymeric carrier. In the case of celecoxib, the E-173 variant of the novel ModE copolymer proved to be a novel polymeric carrier with great potential for application in S-SNEDDS. The presented approach will, therefore, be pursued in future studies to establish S-SNEDDS as an alternative formulation to other amorphous systems.

## 1. Introduction

Poor water solubility is one of the most important reasons for the limited and variable oral bioavailability of drugs [1]. While about 40% of orally administered drugs currently on the market are affected by poor water solubility, this problem is increasing significantly for newer drugs. Among drugs under development, an estimated 75% of drug substances suffer from poor aqueous solubility [2]. Moreover, this proportion is expected to increase further in the future. This clearly demonstrates the importance of developing effective strategies to improve drug solubility when aiming to develop safe and effective oral drug products.

In recent decades, several formulation strategies have been developed that can increase the apparent solubility of poorly water-soluble drugs in gastrointestinal fluids and, provided they are drugs that can readily permeate the intestinal mucosa, also enhance oral bioavailability [3]. The individual formulation approaches used can be very different and usually are directed by the properties of the drug substance to be formulated. For lipophilic drugs, the development of lipid-based drug delivery systems (LBDDS) represents a valuable means of addressing solubility and bioavailability issues. In particular, self-nanoemulsifying drug delivery systems (SNEDDS), which are multicomponent systems containing a lipophilic drug in a mixture of an oil or a lipid and a surfactant or a mixture of surfactants and, optionally also a co-solvent, represent an interesting option to improve the limited water solubility of drugs [4,5,6]. When in contact with gastrointestinal fluids and under the influence of digestive, gastrointestinal motility, SNEDDS spontaneously form transparent nanoemulsions in which the formulated drug is solubilized [7]. The formulations commonly known as SNEDDS are usually liquid formulations, which are also known as Liquid-SNEDDS (L-SNEDDS). L-SNEDDS allow high loading with poorly water-soluble drugs and can be prepared rather quickly and by simple means. However, the fact that they are liquid formulations also presents some disadvantages [5,8]. To obtain administrable dosage forms, L-SNEDDS usually require cost-intensive processing into soft capsules, as can be seen in the example of Neoral*^®^* or Fortovase*^®^* [8,9]. However, this formulation step does not always guarantee a stable dosage form, as L-SNEDDS can interact with and, in the worst case, even leak out of the capsule shell [4,10,11]. In addition, drug precipitation can occur during storage for a variety of reasons. The development of an innovative solid dosage form that preserves the advantages of the L-SNEDDS formulation, but eliminates its disadvantages as much as possible, could hence be beneficial [8,10,12]. Various commonly described manufacturing technologies, such as adsorption to a solid carrier, wet granulation, spray drying, freeze drying, and supercritical fluid processes, can be used to convert L-SNEDDS into Solid-SNEDDS (S-SNEDDS) [4,8,13]. At present, the most common formulation approach currently used to produce S-SNEDDS is the adsorption of L-SNEDDS to a solid carrier [13,14].

Hot melt extrusion (HME) may be another option for S-SNEDDS production [10]. HME is a process that in the pharmaceutical industry has been used for quite some time, mainly for producing solid amorphous dispersions (ASDs) [15,16]. The process is solvent-free, easy to scale up, and can be used for continuous production. Since it is also less time-consuming than many of the other technologies used for S-SNEDDS preparation, it represents an interesting alternative for the development of S-SNEDDS. To date, HME has not been studied for the purpose of S-SNEDDS production. However, very recently first attempts to utilize this technology in the preparation of solid self-microemulsifying drug delivery systems (S-SMEDDS) were reported [10].

A successful HME process requires the input of both thermal and mechanical energy. To enable the conversion of the solid polymeric material into a flowable melt, a process temperature above the glass transition temperature (T_g_) of the respective polymer(s) is required. Due to the thermal input when aiming to formulate S-SNEDDS via HME, the process temperature might be an essential parameter. It will need to be kept below the decomposition temperatures of the individual components in order to prevent changes in material properties, the formation of toxic by-products and the possible loss of active ingredients. In addition to this, the HME of a multicomponent system containing solid and liquid components can be challenging because premature leakage of the liquid components from the matrix during the HME process must be avoided to ensure the correct mixing ratio of the individual components and their uniform distribution in the final solid product. For the polymeric material used in the HME process, in addition to T_g_ [17,18,19], molecular weight (M_w_) [17,20], absorption and binding capacity for oily formulations (e.g., L-SNEDDS), solubilizing capacity [18], decomposition temperature, flexibility, hardness, and brittleness may be important.

The present study aimed to develop drug-loaded S-SNEDDS by the co-extrusion of L-SNEDDS and a polymeric carrier using an HME process. Experiments were performed with celecoxib-, efavirenz-, and fenofibrate L-SNEDDS formulations established in earlier studies [21]. A further objective was to identify a (co)polymer that would enable the preparation of stable S-SNEDDS without impairing the release behavior and storage stability of the L-SNEDDS used and, if possible, even improve them further. In addition to commercially available (co)polymers already used in the field of HME, a particular focus was on the evaluation of different variants of a recently developed aminomethacrylate-based copolymer that differed in M_w_ [22].

## 2. Results and Discussion

### 2.1. L-SNEDDS Composition

The qualitative and quantitative composition of celecoxib-, efavirenz-, and fenofibrate L-SNEDDS formulations prepared by applying the screening approach developed in a preceding study [21] is shown in Table 1. With 25%, particularly for the efavirenz L-SNEDDS, a high drug load was obtained. Drug loads for the celecoxib- and the fenofibrate L-SNEDDS were 17% and 14%, respectively.

All L-SNEDDS formulations shown in Table 1 provided good emulsification properties resulting in small droplet sizes and a narrow PDI (celecoxib L-SNEDDS: 24.4 ± 0.2 nm, 0.11 ± 0.01 [21], efavirenz L-SNEDDS: 36.7 ± 0.3 nm, 0.08 ± 0 and fenofibrate L-SNEDDS: 18.6 ± 0.3 nm, 0.06 ± 0.01 [21]) and thus were selected for subsequent HME processing.

### 2.2. S-SNEDDS Composition and Manufacture

After preparation and characterization of the different L-SNEDDS formulations (Table 1), a variety of S-SNEDDS formulations was prepared from each of the L-SNEDDS formulations and marketed (co)polymers commonly used in the field of solubility enhancement, as well as the different novel ModE copolymer types, referred to as E-173 kDa, E-254 kDa, E-281 kDa, and E-305 kDa according to their average molecular weight. Table 2 shows the L-SNEDDS and drug loads of the individual S-SNEDDS formulations, as well as the process parameters (extrusion temperature, screw speed, and torque) used for their preparation. The extrusion temperature, as well as the screw speed, were chosen individually for each (co)polymer–L-SNEDDS combination based on its melt viscosity when subjected to mechanical and thermal input in the HME process. The torque was recorded during HME for each composition and increased with higher melt viscosities. When selecting suitable extrusion parameters, care was taken to ensure that these resulted in torque values that did not exceed 200 Ncm. Torque values > 200 Ncm typically correlated with a pronounced melt viscosity, which could complicate the processing by clogging the extruder die. Compared to a previous study in which drug-loaded ASDs were prepared and in which the same active ingredients and similar amounts of active ingredients were used [22], S-SNEDDS could be prepared at lower extrusion temperatures than the corresponding ASDs due to the plasticizing effect of the incorporated L-SNEDDS. This facilitated overall processing and resulted in substantially lower torque values than in the preceding study [22].

Affinisol*^®^* HPMC 100 LV was extruded at a lower screw speed than all other (co)polymers, as it would otherwise have clogged the extruder die or led to a loss of the integrity of the polymer when further increasing the extrusion temperature. Individual extrusion temperatures were used for each L-SNEDDS (co)polymer combination, while all extrusion temperatures were in the range of reported extrusion temperatures of the corresponding polymers [2,10,15,16,23].

In order to enable a good comparison of formulations, the same L-SNEDDS load and drug content was targeted for each individual S-SNEDDS formulation. Except for the Soluplus*^®^*-based S-SNEDDS, this could be realized, whereas for reasons of processability, a slightly lower L-SNEDDS load had to be applied when working with Soluplus*^®^*. When co-processed with the Soluplus*^®^* copolymer, an L-SNEDDS load of higher than 20% (celecoxib and fenofibrate) or 16.67% (efavirenz) did not provide a solid strand. Reasons for these limitations could be the lower T_g_ of Soluplus*^®^* compared to all other (co)polymers processed by HME in this study, as well as a possibly insufficient binding capacity for oily formulations. However, since the manufacture of S-SNEDDS via HME is largely unexplored, this can only be speculated since data for comparing the process parameters as well as the properties of obtained S-SNEDDS is lacking.

### 2.3. Thermal Characterization of the Pure Drugs, (Co)Polymers, and S-SNEDDS via DSC Analysis

Immediately after processing, the thermal characterization of celecoxib- (Figure S1a,b), efavirenz- (Figure S2a,b), and fenofibrate S-SNEDDS (Figure S3a,b), as well as the individual (co)polymers, was conducted via DSC. Without exception, the thermograms indicated amorphous properties (no melting peaks) for all S-SNEDDS formulations, irrespective of the L-SNEDDS, drug substances, and (co)polymers used (Table 3 and Figures S1–S3), while the crystalline character of the pure drug substances was revealed by a characteristic endothermic peak in the respective melting range of the individual drug substances (Figures S1–S3). Due to the plasticizing effect of the L-SNEDDS used, in comparison to the corresponding (co)polymers, a lower T_g_ could be measured for all S-SNEDDS (Table 3). The lowest T_g_ (31 °C), which was close to the storage temperature (30 °C) chosen for the stability studies, was determined for the Soluplus*^®^*-based fenofibrate S-SNEDDS. The chosen storage conditions might, therefore, allow for increased movement of the polymer chains, which could result in higher mobility of the respective drug combined with an increased risk of (re-)crystallization and thus result in lower drug release.

### 2.4. Saturation Solubility Studies

The saturation solubility (48 h, 20 °C) of celecoxib, efavirenz, and fenofibrate in water was determined for the pure drugs and all L-SNEDDS and S-SNEDDS (Table 4). Since the three drug compounds exhibit pH-independent solubility under physiological conditions of the gastrointestinal tract, as a first estimate, solubility tests in water were considered sufficient for an initial screening and assessment of the suitability of different (co)polymers for the preparation of S-SNEDDS with the desired performance [21,24]. Very low aqueous solubilities were determined for all pure drug compounds (Table 4).

As can be seen from the results in Table 4, it was possible to significantly (*p* < 0.05) improve the solubility of the model drugs used by preparing L-SNEDDS. The results also indicate that by processing L-SNEDDS to S-SNEDDS, it may be possible to further improve the solubility of the drug compounds. However, whether such an effect is achieved depends very much on the composition of the respective formulation. S-SNEDDS based on all commercial polymers used in the study resulted in improved solubility for all drugs compared to that obtained with the underlying L-SNEDDS formulation. For celecoxib and fenofibrate, the largest solubility-enhancing effect was observed for S-SNEDDS based on Affinisol^®^ HPMC 100 LV, whereas for efavirenz S-SNEDDS, this was the case for the Soluplus*^®^*-based S-SNEDDS (Table 4). The effect of solubility enhancement of the S-SNEDDS prepared with ModE was not as pronounced as for the those based on the marketed (co)polymers. Independent of the drug compound, among the ModE-based S-SNEDDS, those made with E-173 kDa presented with higher drug solubilities than the underlying L-SNEDDS formulation. Particularly with the celecoxib- and the efavirenz S-SNEDDS, there was a clear trend toward decreasing drug solubility with the increasing M_w_ of the polymer, and solubilities were, in several cases, lower than those obtained with the L-SNEDDS formulation. However, with all ModE-based S-SNEDDS, the saturation solubility of all three drug compounds was still tremendously higher than that of the pure drug substances.

One explanation for the limited solubility of celecoxib, efavirenz, and fenofibrate in water when formulated as S-SNEDDS with the ModE copolymer could be the limited water solubility of ModE itself. Based on the observation that ModE does not dissolve properly in water, L-SNEDDS containing any of the above poorly water-soluble active ingredients may not be released to the same extent as in the case of S-SNEDDS based on commercially available, pH-independent, soluble (co)polymers.

### 2.5. Dissolution Studies

To get a first impression of the drug release behavior of L- and S-SNEDDS formulations, dissolution studies of drug-loaded L- and S-SNEDDS were performed in USP apparatus II using 500 mL of 0.1 M hydrochloric acid (HCl) as a dissolution medium. For comparative purposes, corresponding doses of the unprocessed drug substances were also investigated in the same test setup.

**Figure 1:**
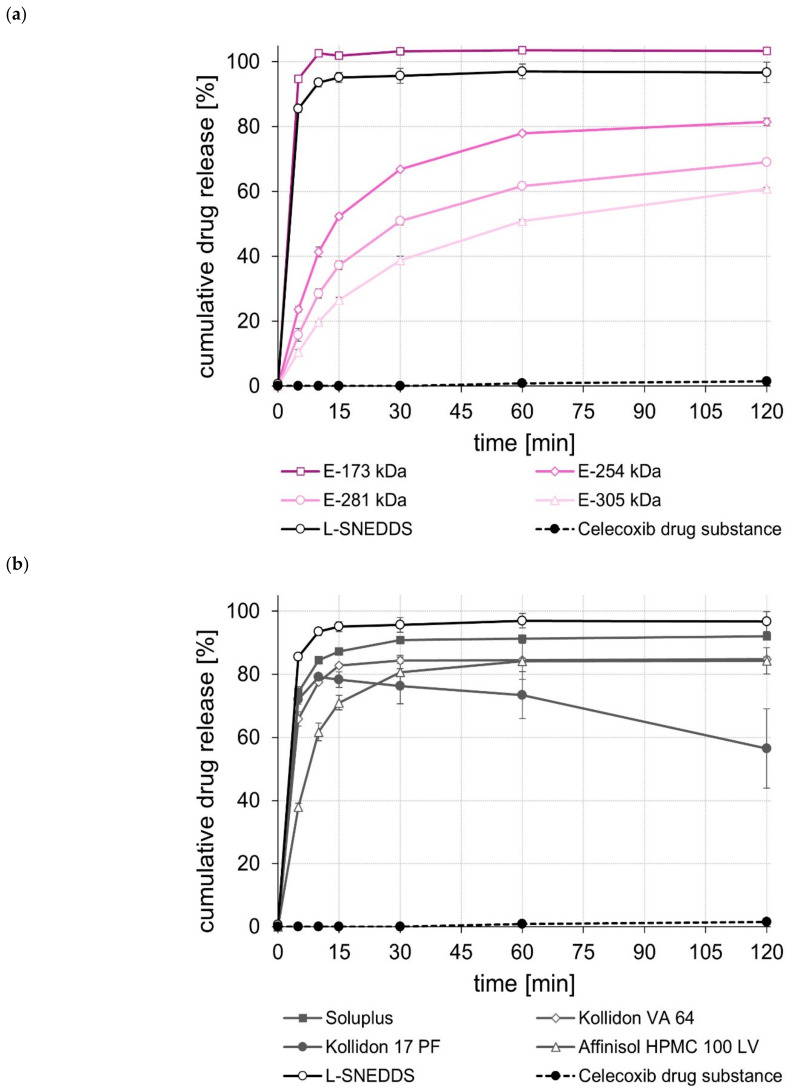
Dissolution profiles of the celecoxib drug substance and celecoxib L- and S-SNEDDS based on ModE (**a**), as well as the celecoxib drug substance and celecoxib L- and S-SNEDDS based on other marketed (co)polymers (**b**) in 500 mL 0.1 M HCl in USP apparatus II. Each value designates the mean ± S.D. (*n* = 3).

**Figure 2:**
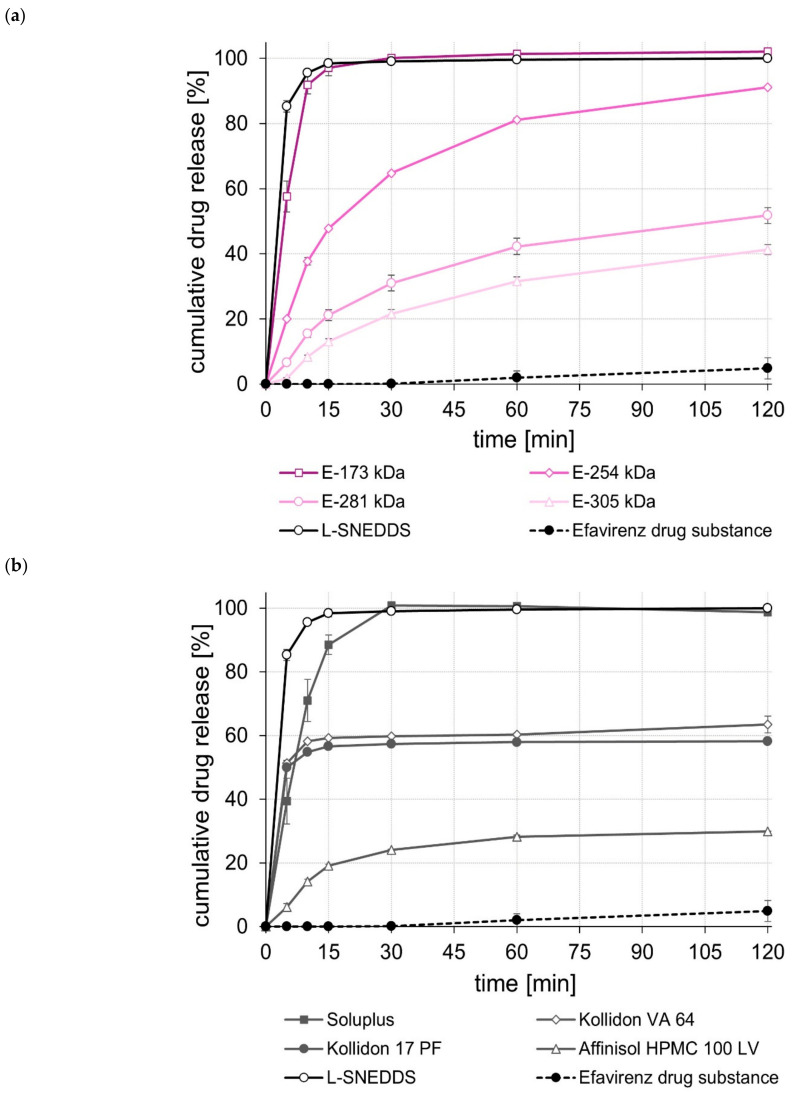
Dissolution profiles of the efavirenz drug substance and efavirenz L- and S-SNEDDS based on ModE (**a**), as well as the efavirenz drug substance and efavirenz L- and S-SNEDDS based on other marketed (co)polymers (**b**) in 500 mL 0.1 M HCl in USP apparatus II. Each value designates the mean ± S.D. (*n* = 3).

**Figure 3:**
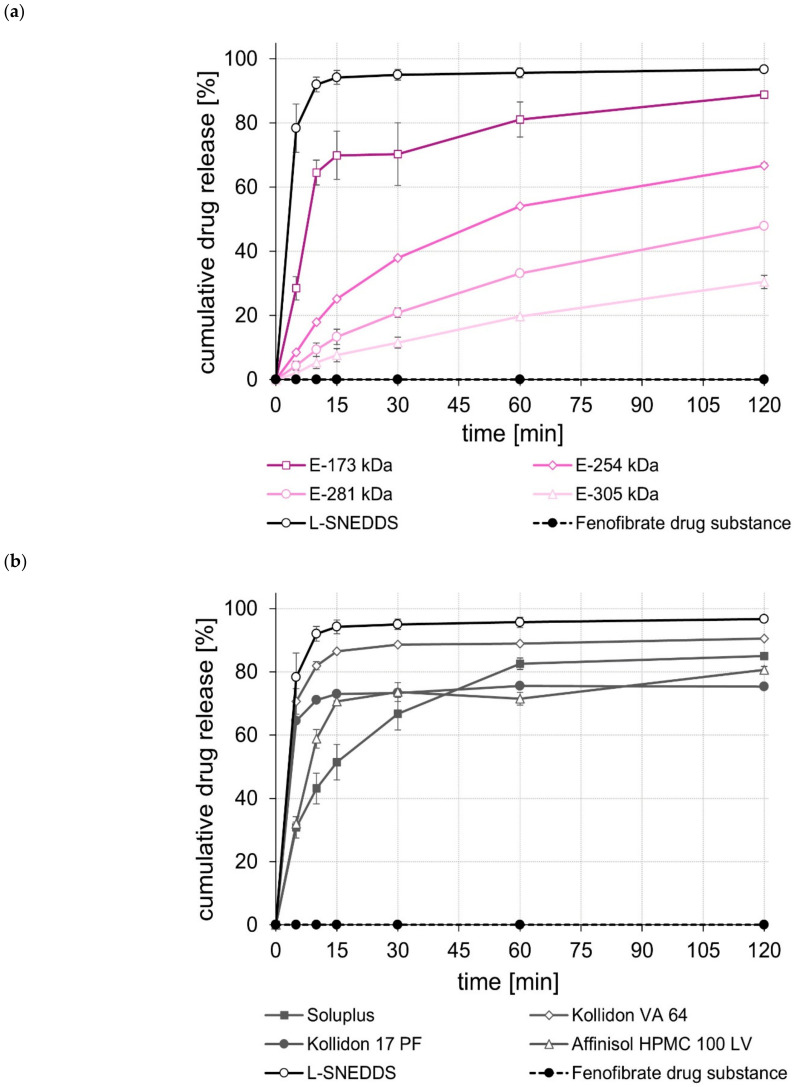
Dissolution profiles of the fenofibrate drug substance and fenofibrate L- and S-SNEDDS based on ModE (**a**), as well as the fenofibrate drug substance and fenofibrate L- and S-SNEDDS based on other marketed (co)polymers (**b**) in 500 mL 0.1 M HCl in USP apparatus II. Each value designates the mean ± S.D. (*n* = 3).

The dissolution of the unprocessed drug substances was very poor, i.e., within the test duration of 120 min, in all cases, less than 5% of the tested dose was released. In contrast, the L-SNEDDS formulations, regardless of whether they contained celecoxib, efavirenz, or fenofibrate (Figure 1, Figure 2 and Figure 3), showed rapid and complete drug release within 15 to 30 min. In the case of celecoxib, S-SNEDDS preparation did not affect the release rate of the L-SNEDDS formulation when the E-173 kDa copolymer was used. (Figure 1a), i.e., drug release was still fast and complete. Similar observations were made for the S-SNEDDS made with the Soluplus*^®^* copolymer (Figure 1b). Within the same time period, the S-SNEDDS formulations prepared with Kollidon*^®^* VA 64, Affinisol*^®^* HPMC 100 LV, and E-254 kDa released about 80% to 85% of the incorporated celecoxib dose, while celecoxib release from all other S-SNEDDS formulations was slower in most cases, with markedly less celecoxib released within 120 min (Figure 1a,b). Similarly, for efavirenz, the drug release performance of the S-SNEDDS made of the E-173 kDa or the Soluplus*^®^* copolymer was fast and complete and similar to that of the L-SNEDDS (Figure 2a,b). Within the same test duration, the S-SNEDDS made of the E-254 kDa showed a slightly lower efavirenz release (about 90% of the dose released) (Figure 2a), whereas all other S-SNEDDS formulations presented with a much lower amount of drug release in this time period (Figure 2a,b). Observations made with the fenofibrate S-SNEDDS were slightly different. Of the S-SNEDDS formulations, those made with Kollidon*^®^* VA 64 presented the best in vitro performance (about 90% of the dose released) (Figure 3b). Within the same time period, approximately the same total amount of fenofibrate (~90%) was released from the E-173 kDa S-SNEDDS, but at a lower release rate (Figure 3a). S-SNEDDS based on Soluplus^®^ (~85%), Affinisol^®^ HPMC 100 LV (~80%), and Kollidon^®^ 17 PF (~76%) also released a large proportion of the contained fenofibrate dose within the test duration of 120 min (Figure 3b). Overall, the amount of fenofibrate released from all S-SNEDDS formulations studied was a little lower than that observed for the underlying L-SNEDDS formulation. In summary, the results of the release studies show that, by using a suitable (co)polymer as well as a suitable manufacturing process, it is possible to convert L-SNEDDS into S-SNEDDS while barely changing the original release behavior.

A comparison of the rate and extent of drug release of the S-SNEDDS prepared from different ModE types showed that a higher M_w_ or a higher T_g_ of the ModE copolymer used resulted in a decrease, both in the rate and extent of celecoxib-, efavirenz-, and fenofibrate release within the test period of 120 min (Figure 1a, Figure 2a and Figure 3a). These observations correlate with those made when ModE was used in the preparation of ASD [22]. The observed effect of M_w_ and T_g_ on drug release performance (dissolution rate of the drug substance was inversely proportional with M_w_ and T_g_ of a copolymer) was also determined by Knopp et al. [25], who studied polyvinylpyrrolidone-based ASDs incorporating the drug substance celecoxib. Further, for these formulations, a decrease in the (co)polymer’s M_w_ was accompanied by decreasing viscosity and increasing flexibility of polymeric chains [25]. These effects are likely to contribute to better solubilization of poorly water-soluble drug substances [25]. Concluding from these observations on the release performance obtained when E-173 kDa was used in the S-SNEDDS technology, it can be assumed that the higher flexibility of the polymer chains in the E-173 kDa copolymer resulted in good solubilization of the drugs celecoxib, efavirenz, and fenofibrate, respectively. As already stated, the trend regarding an improved drug release performance of S-SNEDDS containing ModE with lower M_w_ observed in the present study was in good agreement with that observed in a previous study [22]. In both studies, the drug release of celecoxib-, efavirenz- and fenofibrate formulations with the same drug dose was investigated. Comparing the results, the S-SNEDDS formulations provide significantly (*p* < 0.05) better in vitro release performance than the ASD formulations. This is particularly clear from the example of fenofibrate, where the E-173 kDa ASD formulation released 30% [22] and the corresponding S-SNEDDS formulation almost 90% of the fenofibrate dose within a period of 120 min under the same test conditions.

This first set of dissolution studies was performed using 0.1 M HCl as the dissolution medium to simulate the pH conditions of the fasting stomach, in which the ModE copolymer is also soluble. By not adding artificial surfactants, such as sodium lauryl sulfate or polysorbate 20, to the dissolution medium, which were used in previous studies to screen drug release from formulations containing celecoxib [25,26], efavirenz [15,23], and fenofibrate [16,27,28], worst-case conditions should be established to clearly highlight the effects of the S-SNEDDS technology on drug release. For the same reason, the use of physiologically relevant amounts of bile salts was refrained from. Since the pH-independent solubility of all three agents used in the present series of tests has been demonstrated in previous studies [21,24], it can be assumed that the dissolution behavior of celecoxib, efavirenz, and fenofibrate will be the same or similar in media at other pH values.

Comparison with other dissolution data related to S-SNEDDS prepared by HME is not (yet) possible at this time because of the lack of studies in this field. Silva et al. [10] have recently published preliminary results from a similar study. They chose hydroxypropyl methylcellulose acetate succinate (HPMCAS) for the preparation of S-SMEDDS by HME. However, they did not develop an S-SNEDDS formulation, they used a different active ingredient as well as a different loading level, and the experimental conditions in the release studies performed were also different from those used in the present study. Therefore, if one wants to discuss the formulation’s influence on drug release, it must remain at this point for the corresponding drug when comparing L-SNEDDS, S-SNEDDS, and ASDs.

### 2.6. Stability Studies

#### 2.6.1. Appearance (After Three and Six Months of Storage)

After three months of storage under defined and constant conditions (30 °C/65% RH), the appearance of all L-SNEDDS formulations remained unchanged regardless of the drug substance incorporated. The stored samples of most S-SNEDDS formulations showed no significant agglomeration and could be easily shaken up. Only S-SNEDDS prepared using the copolymer Soluplus^®^ were found to have larger agglomerates in samples containing the active ingredients celecoxib as well as efavirenz. After a further three months, i.e., after a total of six months of storage under the same conditions (30 °C/65% RH), very slight turbidity was observed in the L-SNEDDS. As before, most of the stored S-SNEDDS formulations could be easily shaken up again. Agglomeration was observed for the celecoxib S-SNEDDS prepared using Soluplus^®^ and Kollidon^®^ 17 PF. Larger agglomerates were also evident in efavirenz S-SNEDDS based on the (co)polymers Soluplus^®^, Kollidon^®^ VA 64, and Affinisol^®^ HPMC 100 LV. In the case of fenofibrate S-SNEDDS, agglomeration was observed exclusively with Soluplus^®^-based S-SNEDDS. There was a fairly clear correlation between the agglomeration tendency and the T_g_s of the corresponding S-SNEDDS formulations, which will be discussed in the following section. Overall, the agglomeration tendency was increased when the temperature of the storage conditions and the T_g_ were close to each other.

#### 2.6.2. Thermal Characterization of S-SNEDDS via DSC Analysis (After Six Months of Storage)

Thermal characterization of the pure (co)polymers after six months of storage revealed that the applied storage conditions did not have any significant impact on the T_g_ of the pure (co)polymers (Table 3 and Table 5). Furthermore, although storage conditions affected the T_g_ of all S-SNEDDS, regardless of the drug substance used, these still exhibited an amorphous state when the corresponding thermograms of the first heating cycle were analyzed (Figures S1–S6, Table 3 and Table 5). In some cases, after storage, the T_g_s of S-SNEDDS were even closer to the elevated storage temperature of 30 °C as before storage. Substantial decreases in T_g_ (more than 5 °C below the initial value) were observed for celecoxib S-SNEDDS based on Kollidon^®^ VA 64, efavirenz S-SNEDDS based on Soluplus^®^ and E-305 kDa, and fenofibrate S-SNEDDS prepared using different ModE copolymer types (Table 3 and Table 5). Interestingly, none of the corresponding thermograms showed evidence for crystallization. This may be due, in particular, to the special formulation concept of the S-SNEDDS. The fact that the active ingredients used were completely dissolved in L-SNEDDS before conversion to S-SNEDDS seems to substantially contribute to the stabilization of the amorphous state and the lack of crystallization tendency.

#### 2.6.3. Dissolution Studies after Three and Six Months of Storage

After three months of storage at 30 °C/65% RH, there was hardly any change in the release profiles for the drug-loaded L-SNEDDS. After six months of storage, a trend toward a slightly lower amount of released dose (approx. 91–92% of the applied dose) was observed for all active ingredients used when compared with results obtained from release studies directly after the preparation of the individual drug-loaded L-SNEDDS, which could indicate potential stability issues for the L-SNEDDS under these storage conditions.

**Figure 4:**
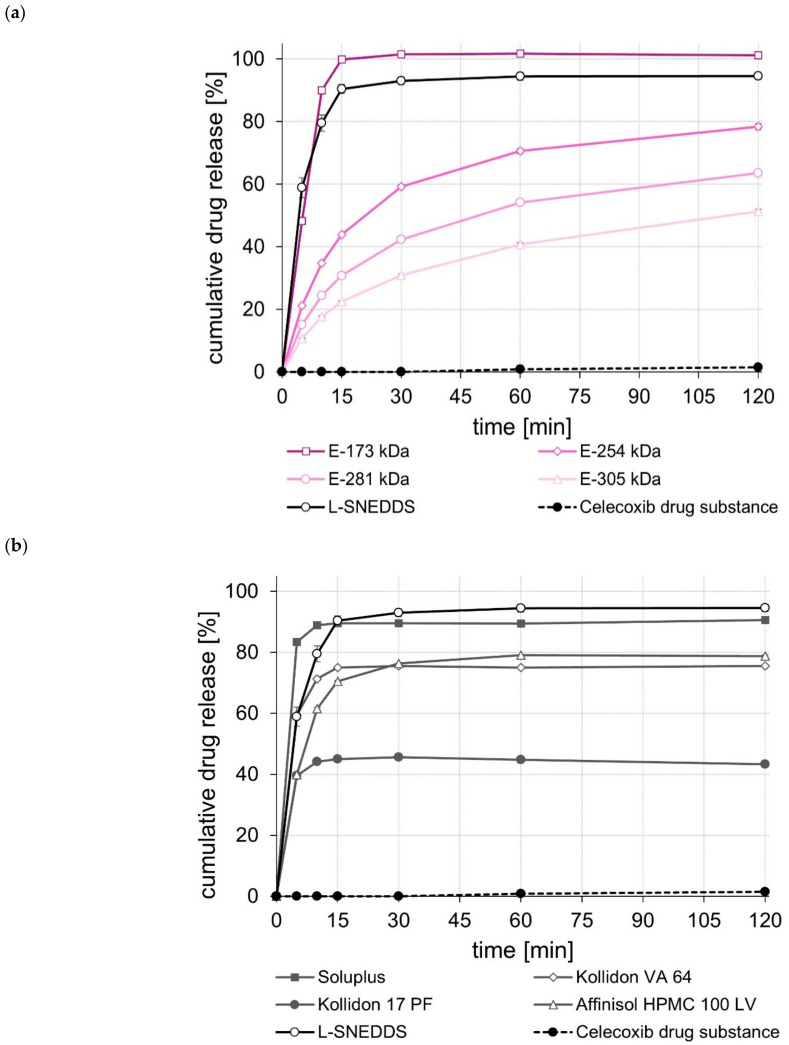
Dissolution profiles of the celecoxib drug substance and celecoxib L- and S-SNEDDS based on ModE (**a**), as well as the celecoxib drug substance and celecoxib L- and S-SNEDDS based on other marketed (co)polymers (**b**) after six months of storage at 30 °C/65% RH in 500 mL 0.1 M HCl in USP apparatus II. Each value designates the mean ± S.D. (*n* = 3).

**Figure 5:**
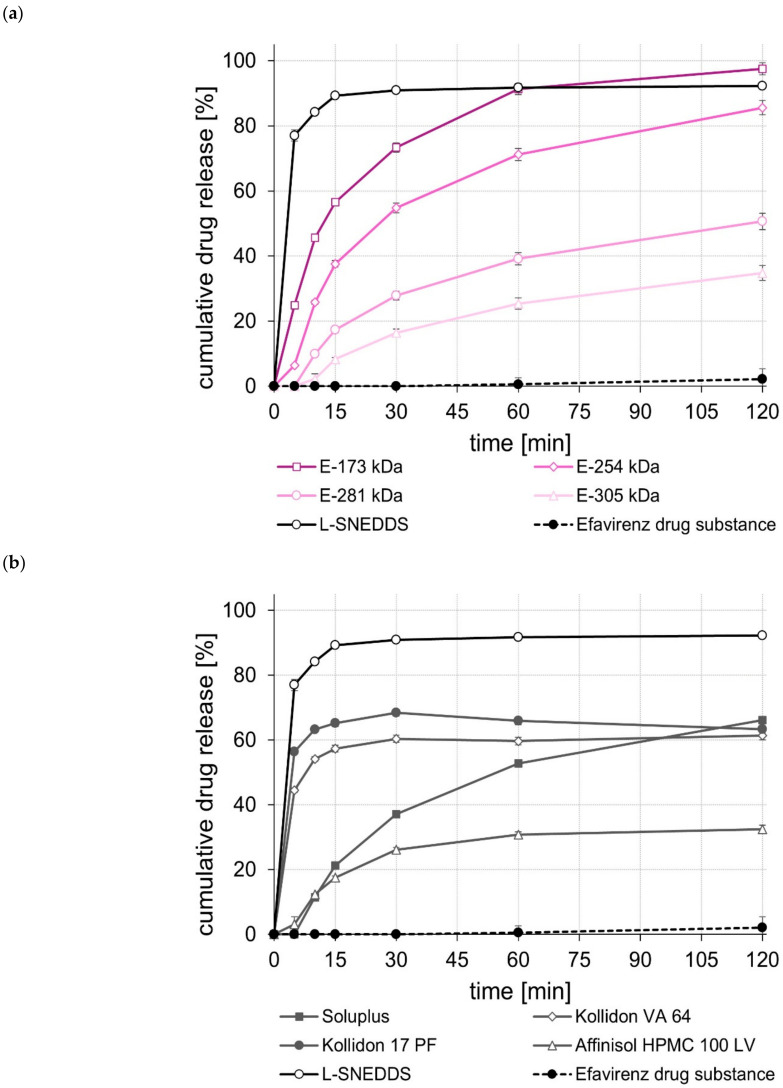
Dissolution profiles of the efavirenz drug substance and efavirenz L- and S-SNEDDS based on ModE (**a**), as well as the efavirenz drug substance and efavirenz L- and S-SNEDDS based on other marketed (co)polymers (**b**) after six months of storage at 30 °C/65% RH in 500 mL 0.1 M HCl in USP apparatus II. Each value designates the mean ± S.D. (*n* = 3).

**Figure 6:**
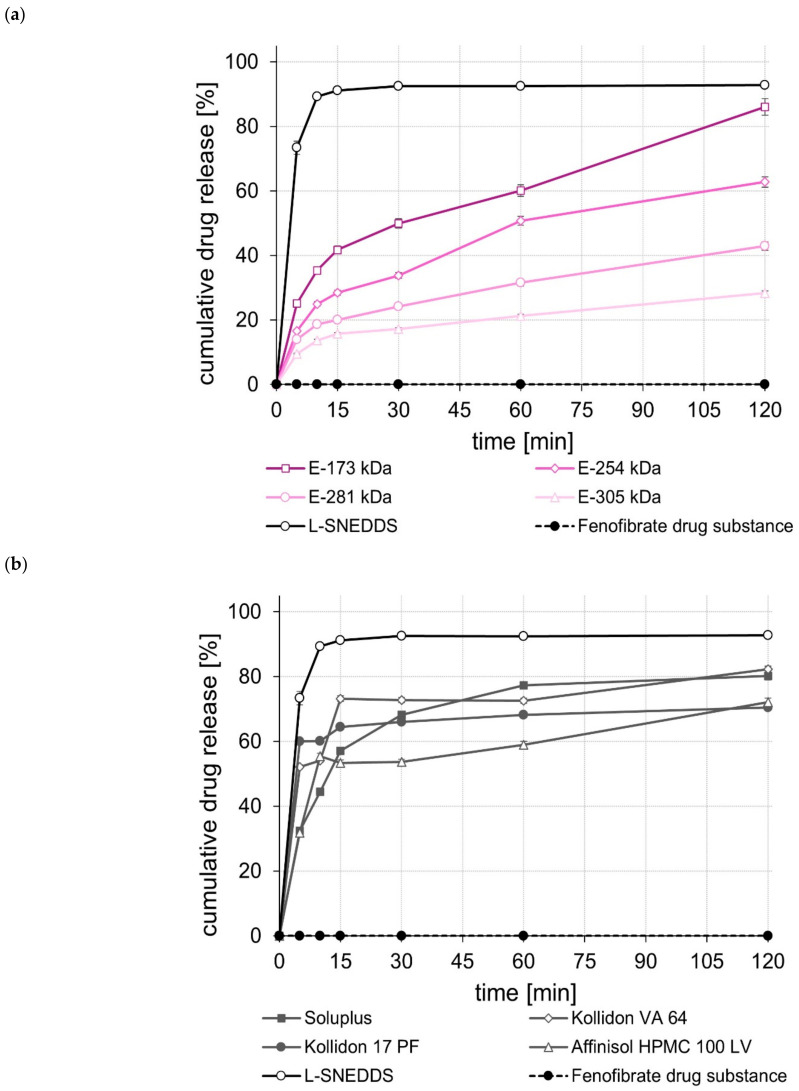
Dissolution profiles of the fenofibrate drug substance and fenofibrate L- and S-SNEDDS based on ModE (**a**), as well as the fenofibrate drug substance and fenofibrate L- and S-SNEDDS based on other marketed (co)polymers (**b**) after six months of storage at 30 °C/65% RH in 500 mL 0.1 M HCl in USP apparatus II. Each value designates the mean ± S.D. (*n* = 3).

After storage of the drug-loaded S-SNEDDS at 30 °C/65% RH for three and six months, most of the S-SNEDDS showed slightly different dissolution performance (Figures S7–S9 and Figure 4, Figure 5 and Figure 6). Whereas the rate and extent of drug release of some of the formulations marginally changed, for some of the S-SNEDDS formulations, a substantial change in drug release performance, particularly with regard to the extent of drug release, could be observed.

For the celecoxib S-SNEDDS, a marked decrease in the total amount of drug released was observed for the Kollidon^®^ 17 PF- and the E-305 kDa-based S-SNEDDS after only three months of storage (Figure S7a,b). For the Kollidon^®^ 17 PF-based S-SNEDDS, this trend continued during the remaining storage time (Figure 4b). For the Kollidon^®^ VA 64-based S-SNEDDS, which showed an almost complete drug release immediately after preparation, both the release rate and the amount of drug released at 120 min after a storage period of six months were noticeably different (Figure 4b). In good agreement with this observation, substantially lower T_g_s were also measured for these S-SNEDDS after six months of storage, suggesting that there may be a correlation between changes in T_g_ and drug release.

For the efavirenz S-SNEDDS, a major decrease in drug release after the test duration of 120 min was noticed for E-305 kDa-based S-SNEDDS after three months of storage (Figure S8a). Similar observations were made for the efavirenz S-SNEDDS prepared with Soluplus^®^ after six months of storage (Figure 5b). As observed in the case of celecoxib-loaded S-SNEDDS, the results obtained with the efavirenz S-SNEDDS formulations based on E-305 kDa and Soluplus^®^ may support the hypothesis of a possible correlation between changes in T_g_ and drug release. Similar to the above-discussed celecoxib S-SNEDDS, these efavirenz S-SNEDDS presented with a pronounced decrease in T_g_ after six months of storage and, correspondingly, provided a significantly (*p* < 0.05) lower amount of drug released after a test duration of 120 min compared to their release performance immediately after manufacture.

Results from dissolution studies with fenofibrate S-SNEDDS based on Soluplus^®^, Kollidon^®^ VA 64, Kollidon^®^ 17 PF, E-281 kDa, and Affinisol^®^ HPMC 100 LV (Figure S9a,b) showed a considerable decrease in fenofibrate release already after three months of storage. However, when these formulations were stored for another three months under the same conditions, this had little effect, and the fenofibrate release profiles remained almost unchanged (Figure 6a,b).

Several S-SNEDDS formulations, particularly many of those prepared with celecoxib or efavirenz, demonstrated good storage stability. The corresponding L-SNEDDS also presented good stability data, especially when stored for three months. When L-SNEDDS were stored for an additional three months, the amount of drug released after 120 min of testing decreased by approximately 8%. A decrease in drug release in the same range, as determined for L-SNEDDS, was also observed for several S-SNEDDS formulations. However, comparing the stability data of the S-SNEDDS with those of the L-SNEDDS collected after six months of storage indicates that, depending on the copolymer used for the preparation of the S-SNEDDS, in some cases, an effect could be obtained with respect to further stabilization of the L-SNEDDS. Overall, the results for the drug release of S-SNEDDS indicate that the type of polymer carrier used for the preparation of S-SNEDDS has a major influence on maintaining the initial release behavior of the L-SNEDDS formulations both immediately after preparation of the S-SNEDDS, but also over a longer storage period under “stress conditions”. Overall, the results of the release studies indicated that fenofibrate appears to be the most challenging model compound to formulate among the three drugs used for the S-SNEDDS formulation approach since fenofibrate-loaded S-SNEDDS showed a markedly reduced drug release already after three months of storage in the case of almost all S-SNEDDS regardless of the polymeric carrier used. In contrast, very good results were obtained for celecoxib, in particular when the new E-173 kDa was used for S-SNEDDS preparation. In this case, an almost unchanged, rapid, and complete release of the investigated drug dose could be observed both shortly after preparation and after a three-month storage period at 30 °C/65% RH.

## 3. Materials and Methods

### 3.1. Materials

Celecoxib (purity of 99.7%) was obtained from Aarti Drugs Ltd. (Mumbai, India), efavirenz (purity of 99.3%) was purchased from Angene International Ltd. (London, UK), and fenofibrate (purity of 99.1%) was obtained from D.K. Pharma Chem PVT Ltd. (Maharashtra, India). Polyoxyethylene (80) sorbitan monooleate (Tween^®^ 80), d-α-Tocopherol polyethylene glycol 1000 succinate (d-TPGS), isopropyl myristate (IPM-100), and polyoxyethylene (23) lauryl ether (Brij^®^ 35) were purchased from Sigma Aldrich Chemie GmbH (Steinheim, Germany). Lauroyl polyoxyl-32 glycerides (Gelucire^®^ 44/14) and diethylene glycol monoethyl ether (Transcutol^®^ HP) were kindly donated by Gattefossé S.A.S (Saint Priest, France). Medium-chain triglycerides (Miglyol^®^ 812) were obtained from Caesar & Loretz GmbH (Hilden, Germany). Polyvinyl caprolactam–polyvinyl acetate–polyethylene glycol graft copolymer (Soluplus^®^, M_w_ = 90,000–140,000 g/mol), polyvinylpyrrolidone-polyvinyl acetate copolymer (Kollidon^®^ VA 64, M_w_ = 45,000–70,000 g/mol) and polyvinylpyrrolidone (Kollidon^®^ 17 PF, M_w_ = 7000–11,000 g/mol) were purchased from BASF SE (Ludwigshafen, Germany). Hydroxypropyl methylcellulose (Affinisol^®^ HPMC 100 LV, M_w_ = 179,000 g/mol) was provided by Dow Chemical Company (Schwalbach am Taunus, Germany). Dimethylaminopropyl methacrylamide-butyl methacrylate-methyl methacrylate copolymer (2:1:1, ratios by weight), a modified Eudragit^®^ E copolymer (ModE) in different M_w_s, i.e., E-173 kDa, E-254 kDa, E-281 kDa, and E-305 kDa, is an in-house product of Evonik Operations GmbH (Darmstadt, Germany) and was synthesized as described in [22]. All other chemicals and solvents were of analytical grade and purchased commercially.

### 3.2. Methods

#### 3.2.1. Development and Preparation of L-SNEDDS

L-SNEDDS incorporating the drug substances celecoxib, efavirenz, or fenofibrate were developed according to a systematic screening approach established by Schmied et al. [21]. The optimal excipient composition and mixing ratio for L-SNEDDS resulted from meeting the following self-imposed specifications: After the dispersion of L-SNEDDS in water, a nanoemulsion with a droplet size < 50 nm, a PDI < 0.15, and transmission of >99% was supposed to result [21]. It should be noted here that these limits were deliberately chosen and very strict but explicitly self-imposed and did not represent regulatory quality criteria.

#### 3.2.2. Preparation of S-SNEDDS via HME

Following their preparation and characterization, drug-loaded L-SNEDDS were co-extruded with different (co)polymers in individual HME processes. Marketed (co)polymers already established for the purpose of solubility enhancement, such as Soluplus*^®^*, Kollidon*^®^* VA 64, Kollidon*^®^* 17 PF, and Affinisol*^®^* HPMC 100 LV, as well as various ModE copolymers with different M_w_ were used as polymeric carriers. Extrudates were prepared as follows: First, 15 g of a mixture of each of the individual (co)polymers and an L-SNEDDS formulation containing one of the poorly soluble model compounds was prepared at a predetermined mixing ratio by mixing the components in a 100-mL jar sealed with a screw cap for 10 min using a turbula mixer (TURBULA*^®^*, WAB Group, Nidderau, Germany). Subsequently, this mixture was processed into S-SNEDDS by HME using a co-rotating HAAKE MiniLab twin screw extruder with a conical screw design (Thermo Fisher Scientific, Dreieich, Germany). The HME process was defined by the set parameters screw speed and process temperature as well as by the torque recorded during the process. The die diameter was 2 mm, and the strand leaving the extruder was allowed to cool during transport via a conveyor belt before it was finally ground to coarse granules by a small chopper with a rotating metal gear. These coarse granules were then pulverized using a ZM 200 ultra-centrifugal mill from Retsch GmbH (Haan, Germany) (mesh size: 0.25 mm). The obtained powders were used for all subsequent experiments.

#### 3.2.3. Differential Scanning Calorimetry (DSC) Analysis

All S-SNEDDS formulations were analyzed via DSC (DSC 3+ (DSC-HC01), Mettler Toledo, Giessen, Germany) to determine whether the incorporated drug was in the amorphous (glass transition) or crystalline (melting/crystallization peak) state. The DSC method used was the same as in a preceding study [22]. Briefly, a 5–10 mg sample was weighed into a small aluminum pan that was cold sealed with a perforated lid and exposed to a heating–cooling–heating cycle in a temperature range of 0 to 200 °C. A nitrogen flow of 50 mL/min was applied while running the experiments. The heating and cooling rates were both set at 10 °C/min. The melting point of the pure drug substances, as well as the glass transition temperature of the individual (co)polymers were investigated. For all analyzed samples, T_g_ was taken from the thermogram obtained from the second heating cycle, and the indicated value represents the mean of *n* = 3.

#### 3.2.4. Saturation Solubility Assessments

The saturation solubility of celecoxib, efavirenz, and fenofibrate in water was studied for the pure drug substances and the corresponding L-SNEDDS and S-SNEDDS immediately after preparation. For this purpose, about 25 mg of the drug substance or an amount of S-SNEDDS equivalent to this amount was added to a volume of 25 mL of distilled water and stirred (100 rpm) at a controlled temperature of 20 °C for 48 h. The resulting suspensions were filtered via a 0.22 µm polytetrafluoroethylene (PTFE) membrane filter (25 mm diameter) from Global Biomed Scientific (Forest, VA, USA). The suitability of the filter material was validated for each of the active ingredients before use. All filtrates were diluted in a specific ratio with acetonitrile before quantification of the amount of dissolved celecoxib, efavirenz, and fenofibrate by high-performance liquid chromatography (HPLC).

#### 3.2.5. Dissolution Studies

Dissolution experiments were conducted in triplicate with 25 mg drug substance or an equivalent amount of L-SNEDDS or S-SNEDDS using USP apparatus II (DT 800 LH, ERWEKA GmbH, Langen, Germany). The paddle speed was set to 100 rpm to avoid coning effects, and all experiments were performed in 500 mL of 0.1 M hydrochloric acid at 37 ± 0.5 °C. The test duration was 120 min. All samples were withdrawn via a fraction collector, equipped with cannula filters of 10 µm pore size and, after sampling, were manually diluted in a 1:1 ratio (*v*:*v*) with acetonitrile before HPLC analysis.

#### 3.2.6. HPLC Analysis

An Agilent 1260 Infinity HPLC system was used for the quantification of the different model drug substances. The system consisted of a quaternary pump (G1311B), an autosampler (G1329B), a column oven (G1316A), and a UV detector (G1314C), all from Agilent Technologies (Frankfurt am Main, Germany). The HPLC methods used to quantify celecoxib, efavirenz, and fenofibrate were the same as described by Schmied et al. [22] and, prior to use, had been validated according to USP requirements.

##### HPLC Method for Celecoxib

The separation of all samples containing celecoxib was achieved using a Knauer Nucleosil 100-7 C18 (125 × 4.6 mm, 7 µm) column maintained at 40 °C. The mobile phase consisted of an acetonitrile:water:triethylamine mixture (300:300:0.9 *v*/*v*), adjusted to pH 3.00 with phosphoric acid. The flow rate was set to 1.8 mL/min. An injection volume of 5 µL was applied and celecoxib was detected at 254 nm. In the concentration range of 0.13–542 µg/mL, the analytical curve was linear (r^2^ = 0.999995). The method was found to be accurate (100.2–102.1%) and precise (CV 2.46%) with a quantification limit of 0.05 µg/mL. The total run time was 7 min [22].

##### HPLC Method for Efavirenz

Separation of the samples containing efavirenz was achieved on a Symmetry 300 C18 (250 × 4.6 mm, 5 µm) column maintained at 22 °C. The mobile phase consisted of an acetonitrile:buffer solution (disodium hydrogen phosphate/phosphoric acid adjusted to pH 3.60) mixture (290:210 *v*/*v*). The flow rate was set to 1.5 mL/min. An injection volume of 20 µL was applied, and efavirenz was detected at 247 nm. In the concentration range of 0.13–515 µg/mL, the analytical curve was linear (r^2^ = 0.999894). The method was found to be accurate (101.4–103.0%) and precise (CV 4.05%) with a quantification limit of 0.05 µg/mL. The total run time was 10 min [22].

##### HPLC Method for Fenofibrate

The separation of all samples containing fenofibrate was achieved using a Symmetry 300 C18 (150 × 4.6 mm, 5 µm) column maintained at 22 °C. The mobile phase consisted of an acetonitrile:water mixture (70:30 *v*/*v*), adjusted to pH 2.50 with phosphoric acid. The flow rate was set to 2.0 mL/min. An injection volume of 20 µL was applied, and fenofibrate was detected at 286 nm. In the concentration range of 0.13–526 µg/mL, the analytical curve was linear (r^2^ = 0.999992). The method was found to be accurate (101.2–101.4%) and precise (CV 2.42%) with a quantification limit of 0.05 µg/mL. The total run time was 7 min [22].

For all HPLC methods, the selectivity for the respective drug substances was determined (formulation excipients), and no interference was observed at the retention time of the specific drug. Moreover, the peak area of the drug did not change in the presence of all excipients used in the study.

#### 3.2.7. Stability Studies

A quantity of approximately 10 g of each L-SNEDDS and S-SNEDDS formulation was added to a 30 mL amber glass jar, closed with a screw cap, and stored at constant and controlled conditions (30 °C/65% RH) in a climatic chamber (Model KBF 720, Binder GmbH, Tuttlingen, Germany) for six months. After three and six months, S-SNEDDS were first visually inspected to check whether they could be easily fluffed up again or stuck together (formation of larger agglomerates), whereas L-SNEDDS were analyzed for separation of their constituents and precipitation of the incorporated drug substance. Subsequently, all formulations were subjected to dissolution experiments. In addition, after six months of storage, DSC analyses were performed with the S-SNEDDS formulations. All results obtained in the stability studies were compared with those obtained immediately after manufacture.

#### 3.2.8. Data Analysis

All reported data originated from at least three independent experiments. Significance tests were conducted with SigmaPlot 14.0 from Systat Software GmbH (Erkrath, Germany) using a one-way analysis of variance (ANOVA) followed by the Holm–Sidak test. Significances are indicated with *p* < 0.05 in brackets.

## 4. Conclusions

In the present study, the preparation of S-SNEDDS from L-SNEDDS and polymeric carrier materials using HME was successfully established as a novel technology concept for the preparation of solid lipid-based formulations for improving the solubility of poorly water-soluble drugs. In particular, for the model drug celecoxib, S-SNEDDS could be developed that maintained the rapid and complete drug release of the underlying L-SNEDDS even over an extended storage period. Overall, the data obtained in the study indicate that the presented S-SNEDDS approach is very promising, provided one combines L-SNEDDS with a suitable polymeric carrier. In the case of celecoxib, the E-173 variant of the new ModE copolymer was revealed to be a novel polymeric carrier with potential for application in S-SNEDDS. Overall, the results obtained in the present study indicate that the presented S-SNEDDS formulation approach represents a novel alternative for the development of solid oral dosage forms for poorly soluble drug candidates. It is believed that this formulation approach also offers great potential for the development of stable solid formulations with improved oral bioavailability for other poorly soluble drug candidates. It merits, therefore, strong pursuit, with particular emphasis on experiments that provide more clarity on the expected in vivo behavior. Overall, therefore, it is likely to see more research addressing this formulation approach in the near future.

## Figures and Tables

**Table 1:** Composition of Liquid-SNEDDS (L-SNEDDS) incorporating celecoxib, efavirenz or fenofibrate.

Drug Substance	Miglyol^®^ 812 (%)	Tween^®^ 80 (%)	Gelucire^®^ 44/14 (%)	d-TPGS (%)	IPM-100(%)	Transcutol^®^ HP (%)	Brij^®^ 35 (%)	Drug (%)
Celecoxib	27.64	45.52	4.15	5.69	-	-	-	17.00
Efavirenz	-	23.94	-	2.99	19.45	28.42	-	25.20
Fenofibrate	17.20	50.16	-	10.04	-	-	8.60	14.00

**Table 2:** Composition and hot melt extrusion process parameters of Solid-SNEDDS (S-SNEDDS) incorporating celecoxib/efavirenz/fenofibrate.

Polymer	L-SNEDDS Load (%)	Drug Load (%)	Extrusion Temperature (°C)	Screw Speed (rpm)	Torque (Ncm)
Soluplus^®^	20/16.67/20	3.4/4.2/2.8	130/130/130	200	50/40/45
Kollidon^®^ VA 64	30/25/30	5.1/6.3/4.2	150/150/150	200	45/45/35
Kollidon^®^ 17 PF	30/25/30	5.1/6.3/4.2	170/170/170	200	40/40/35
E-173 kDa	30/25/30	5.1/6.3/4.2	150/150/150	200	75/65/50
E-254 kDa	30/25/30	5.1/6.3/4.2	150/150/155	200	75/70/70
E-281 kDa	30/25/30	5.1/6.3/4.2	155/155/150	200	70/65/75
E-305 kDa	30/25/30	5.1/6.3/4.2	155/155/155	200	60/90/85
Affinisol^®^ HPMC 100 LV	30/25/30	5.1/6.3/4.2	160/160/160	100	85/120/95

**Table 3:** Glass transition temperature (T_g_) of pure (co)polymers and S-SNEDDS incorporating celecoxib, efavirenz, or fenofibrate. Each value designates the mean ± S.D. (*n* = 3).

	T_g_ (°C)			
	Polymer	Celecoxib S-SNEDDS	Efavirenz S-SNEDDS	Fenofibrate S-SNEDDS
Soluplus^®^	70 ± 0	39 ± 2	42 ± 2	31 ± 2
Kollidon^®^ VA 64	107 ± 1	67 ± 3	43 ± 0	47 ± 1
Kollidon^®^ 17 PF	136 ± 3	88 ± 1	60 ± 1	74 ± 2
E-173 kDa	77 ± 1	43 ± 0	38 ± 1	48 ± 0
E-254 kDa	85 ± 2	44 ± 1	37 ± 1	41 ± 0
E-281 kDa	89 ± 0	45 ± 2	38 ± 0	48 ± 1
E-305 kDa	91 ± 1	42 ± 0	42 ± 2	44 ± 1
Affinisol^®^ HPMC 100 LV	103 ± 2	45 ± 1	43 ± 2	36 ± 0

**Table 4:** Saturation solubility (48 h) of celecoxib, efavirenz, and fenofibrate pure drug substances, L-SNEDDS, and S-SNEDDS in water at 20 °C. Each value designates the mean ± S.D. (*n* = 3).

	Saturation Solubility (µg/mL)		
	Celecoxib	Efavirenz	Fenofibrate
Drug substance	0.6 ± 0.1	0.7 ± 0	0.1 ± 0
L-SNEDDS	31.3 ± 1.2	34.7 ± 2.4	17.4 ± 1.8
Soluplus^®^ S-SNEDDS	140 ± 0.9	347 ± 2.3	70 ± 0.4
Kollidon^®^ VA 64 S-SNEDDS	252 ± 1.3	99 ± 0.9	144 ± 1.1
Kollidon^®^ 17 PF S-SNEDDS	197 ± 0.6	226 ± 1.4	125 ± 0.4
E-173 kDa S-SNEDDS	87 ± 0.8	46 ± 0.2	60 ± 0.5
E-254 kDa S-SNEDDS	50 ± 0.1	18 ± 1.0	47 ± 0.3
E-281 kDa S-SNEDDS	26 ± 0.2	16 ± 0.5	43 ± 0.4
E-305 kDa S-SNEDDS	21 ± 0.1	8 ± 0.4	50 ± 0.2
Affinisol^®^ HPMC 100 LV S-SNEDDS	423 ± 6.1	281 ± 3.1	314 ± 3.6

**Table 5:** Glass transition temperature (T_g_) of the pure (co)polymers and S-SNEDDS incorporating celecoxib, efavirenz, or fenofibrate after six months of storage. Each value designates the mean ± S.D. (*n* = 3).

	T_g_ (°C)			
	Polymer	Celecoxib S-SNEDDS	Efavirenz S-SNEDDS	Fenofibrate S-SNEDDS
Soluplus^®^	69 ± 1	39 ± 2	32 ± 2	29 ± 1
Kollidon^®^ VA 64	105 ± 1	60 ± 2	38 ± 1	47 ± 0
Kollidon^®^ 17 PF	135 ± 0	88 ± 1	59 ± 2	75 ± 1
E-173 kDa	77 ± 1	45 ± 1	36 ± 0	38 ± 1
E-254 kDa	83 ± 0	39 ± 1	35 ± 1	33 ± 0
E-281 kDa	88 ± 1	44 ± 0	33 ± 1	32 ± 1
E-305 kDa	91 ± 1	46 ± 2	35 ± 0	30 ± 0
Affinisol^®^ HPMC 100 LV	69 ± 1	39 ± 2	32 ± 2	29 ± 1

## Data Availability

Data is contained within the article and Supplementary Materials.

## Supplementary Materials

The following supporting information can be downloaded at: https://www.mdpi.com/article/10.3390/ph15091135/s1. Figure S1. DSC thermograms of celecoxib, ModE copolymers, and the corresponding S-SNEDDS (a), celecoxib and other marketed (co)polymers used in the study, and the corresponding S-SNEDDS (b); Figure S2. DSC thermograms of efavirenz, ModE copolymers, and the corresponding S-SNEDDS (a), efavirenz and other marketed (co)polymers used in the study, and the corresponding S-SNEDDS (b); Figure S3. DSC thermograms of fenofibrate, ModE copolymers, and the corresponding S-SNEDDS (a), fenofibrate and other marketed (co)polymers used in the study, and the corresponding S-SNEDDS (b); Figure S4. DSC thermograms (after six months of storage at 30 °C/65% RH) of celecoxib, ModE copolymers, and the corresponding S-SNEDDS (a), celecoxib and other marketed (co)polymers used in the study, and the corresponding S-SNEDDS (b); Figure S5. DSC thermograms (after six months of storage at 30 °C/65% RH) of efavirenz, ModE copolymers, and the corresponding S-SNEDDS (a), efavirenz and other marketed (co)polymers used in the study, and the corresponding S-SNEDDS (b); Figure S6. DSC thermograms (after six months of storage at 30 °C/65% RH) of fenofibrate, ModE copolymers, and the corresponding S-SNEDDS (a), fenofibrate and other marketed (co)polymers used in the study, and the corresponding S-SNEDDS (b); Figure S7. Dissolution profiles of celecoxib drug substance and celecoxib L- and S-SNEDDS based on ModE (a), as well as celecoxib drug substance and celecoxib L- and S-SNEDDS based on other marketed (co)polymers (b) (after three months of storage at 30 °C/65% RH) in 500 mL 0.1 M HCl in USP apparatus II. Each value designates the mean ± S.D. (*n* = 3); Figure S8. Dissolution profiles of efavirenz drug substance and efavirenz L- and S-SNEDDS based on ModE (a), as well as efavirenz drug substance and efavirenz L- and S-SNEDDS based on other marketed (co)polymers (b) (after three months of storage at 30 °C/65% RH) in 500 mL 0.1 M HCl in USP apparatus II. Each value designates the mean ± S.D. (*n* = 3); Figure S9. Dissolution profiles of fenofibrate drug substance and fenofibrate L- and S-SNEDDS based on ModE (a), as well as fenofibrate drug substance and fenofibrate L- and S-SNEDDS based on other marketed (co)polymers (b) (after three months of storage at 30 °C/65% RH) in 500 mL 0.1 M HCl in USP apparatus II. Each value designates the mean ± S.D. (*n* = 3).
