# Supplementary material for: Preparation of Solid Self-Nanoemulsifying Drug Delivery Systems (S-SNEDDS) by Co-Extrusion of Liquid SNEDDS and Polymeric Carriers—A New and Promising Formulation Approach to Improve the Solubility of Poorly Water-Soluble Drugs
Source: Pharmaceuticals (Basel). 2022 Sep 11;15(9):1135. doi: 10.3390/ph15091135 (PMC9505398; doi:10.3390/ph15091135)
Supplement: Supplementary file 1 [file pharmaceuticals-15-01135-s001.zip › pharmaceuticals-1854715-supplementary.pdf]

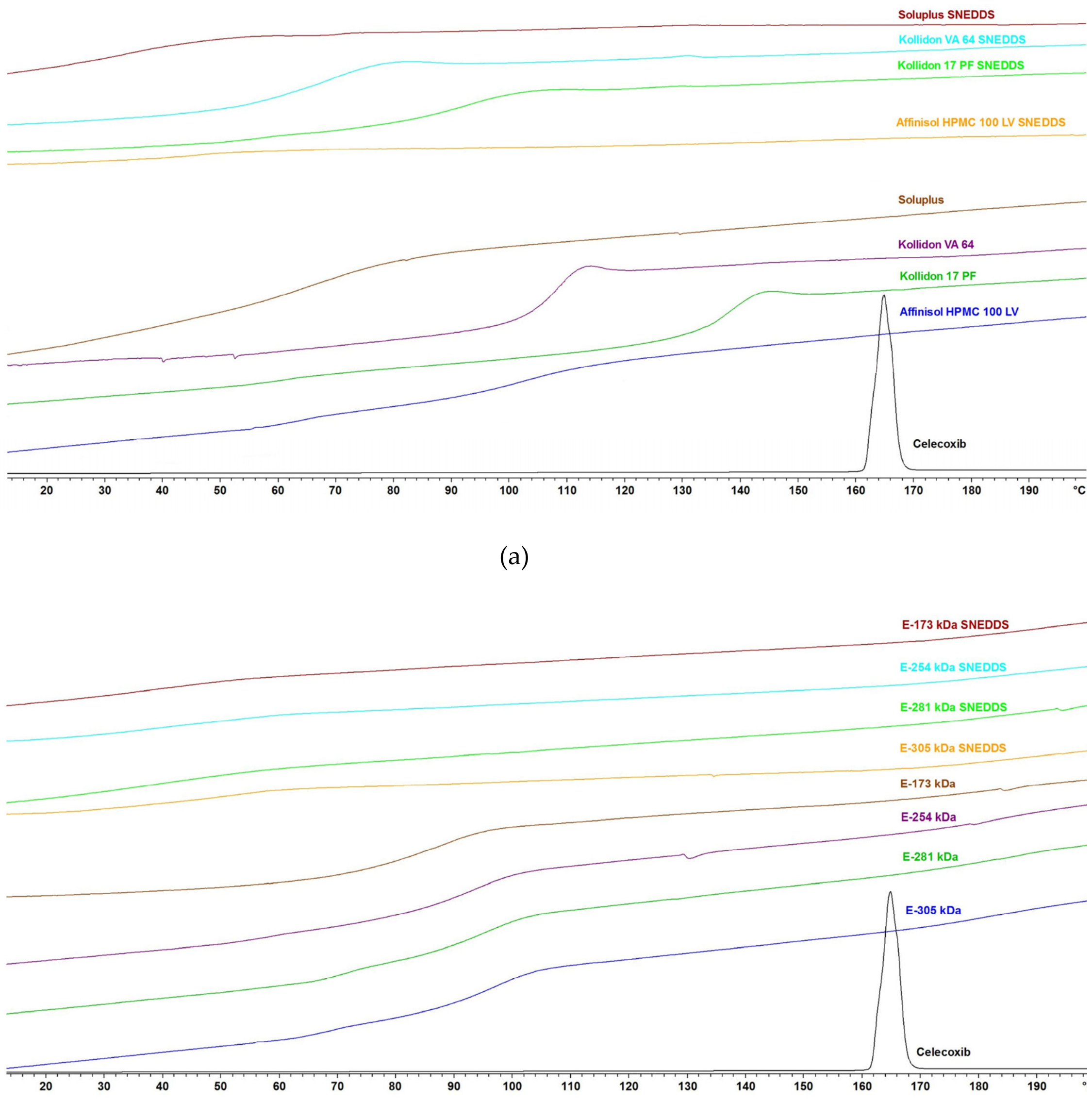

(a)

(b)

Figure S1. DSC thermograms of celecoxib, ModE copolymers, and the corresponding S-SNEDDS (a), celecoxib and other marketed (co)polymers used in the study, and the corresponding S-SNEDDS (b).

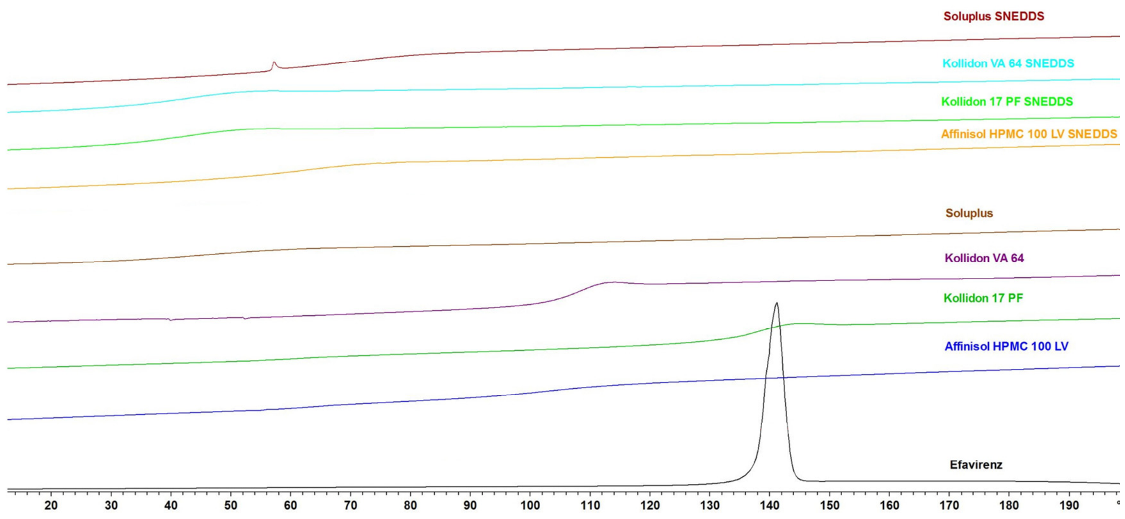

(a)

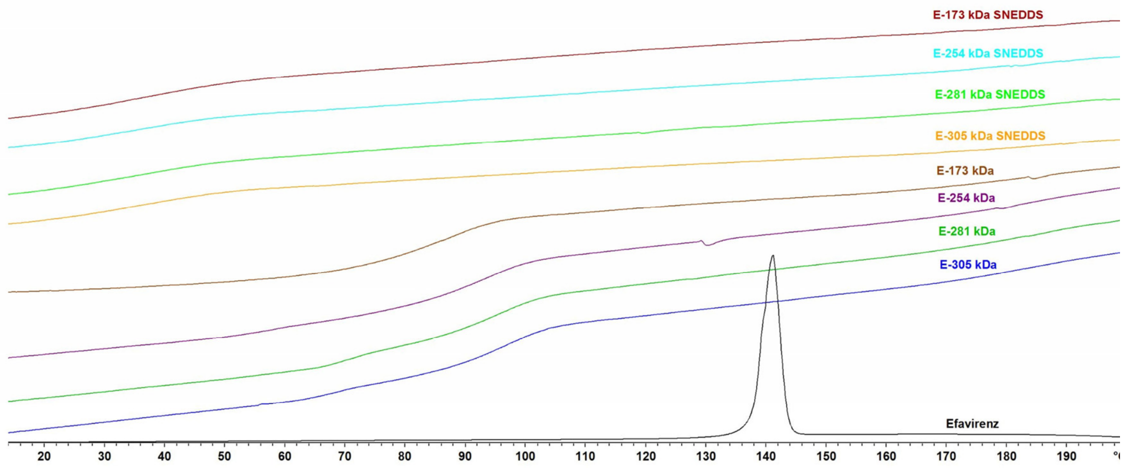

(b)

Figure S2. DSC thermograms of efavirenz, ModE copolymers, and the corresponding S-SNEDDS (a), efavirenz and other marketed (co)polymers used in the study, and the corresponding S-SNEDDS (b)

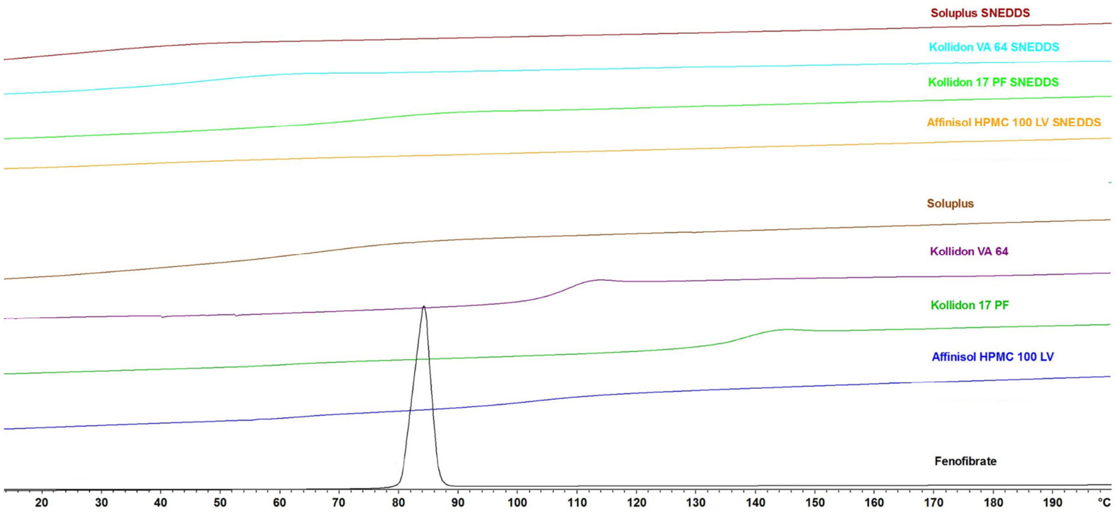

(a)

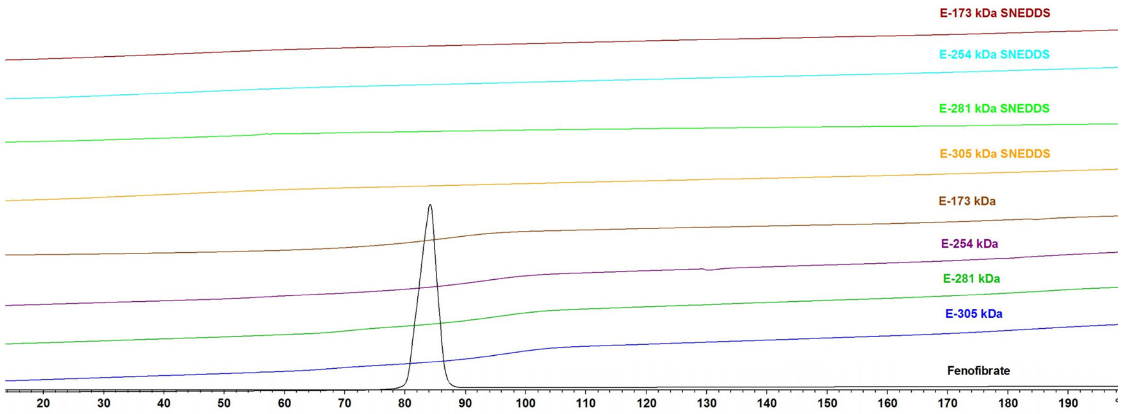

(b)

Figure S3. DSC thermograms of fenofibrate, ModE copolymers, and the corresponding S-SNEDDS (a), fenofibrate and other marketed (co)polymers used in the study, and the corresponding S-SNEDDS (b).

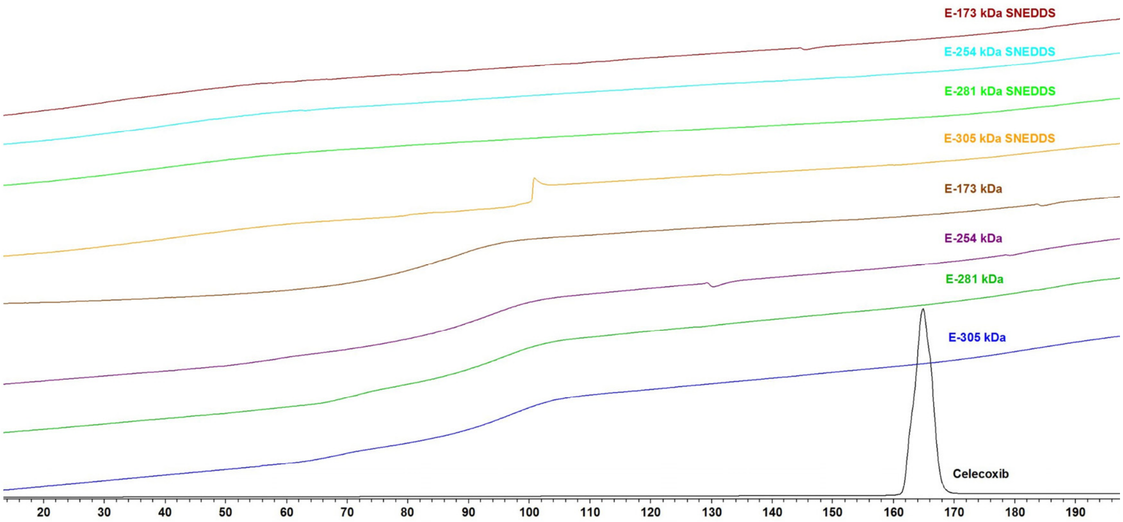

(a)

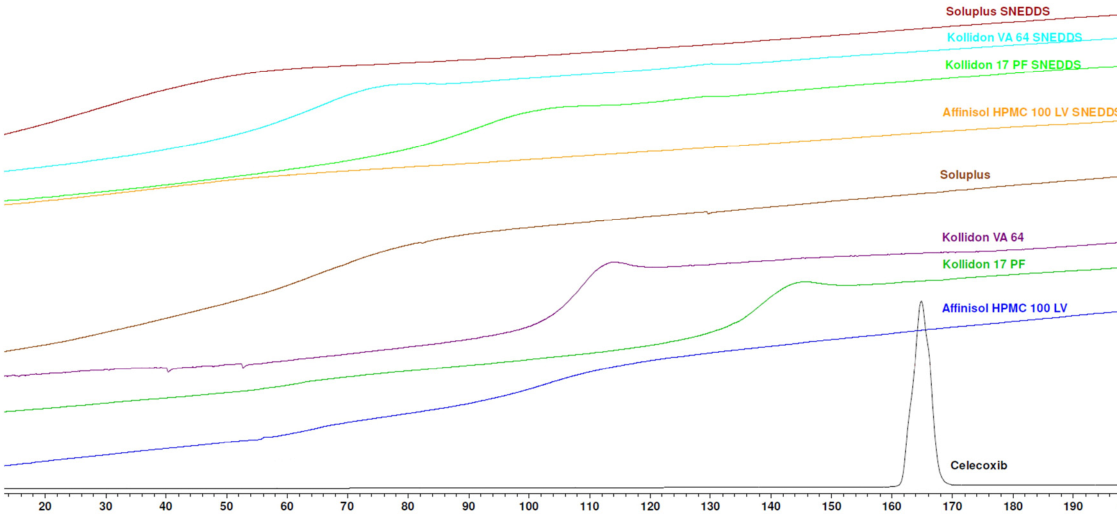

(b)

Figure S4. DSC thermograms (after six months of storage at 30 °C/65% RH) of celecoxib, ModE copolymers, and the corresponding S-SNEDDS (a), celecoxib and other marketed (co)polymers used in the study, and the corresponding S-SNEDDS (b).

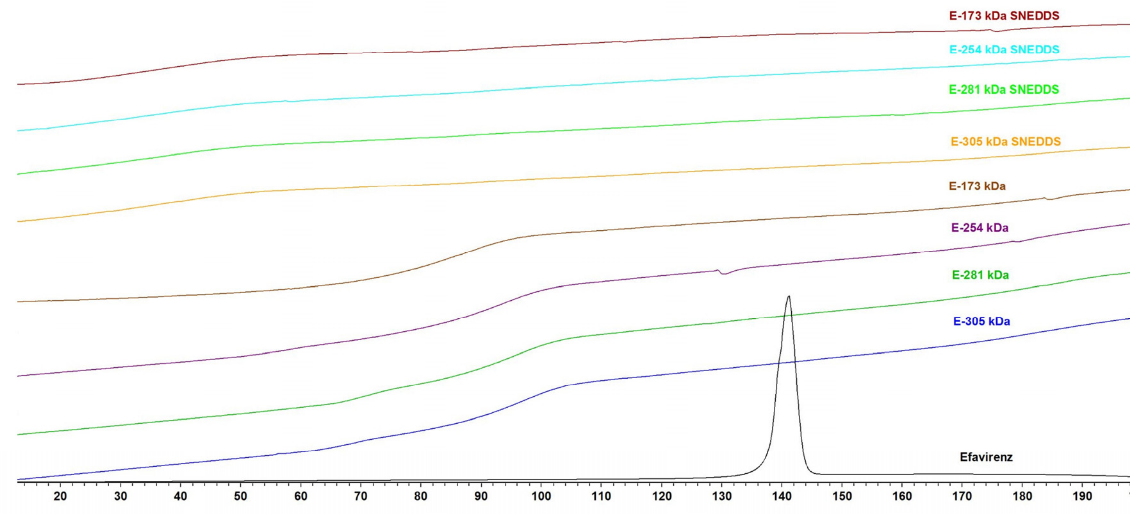

(a)

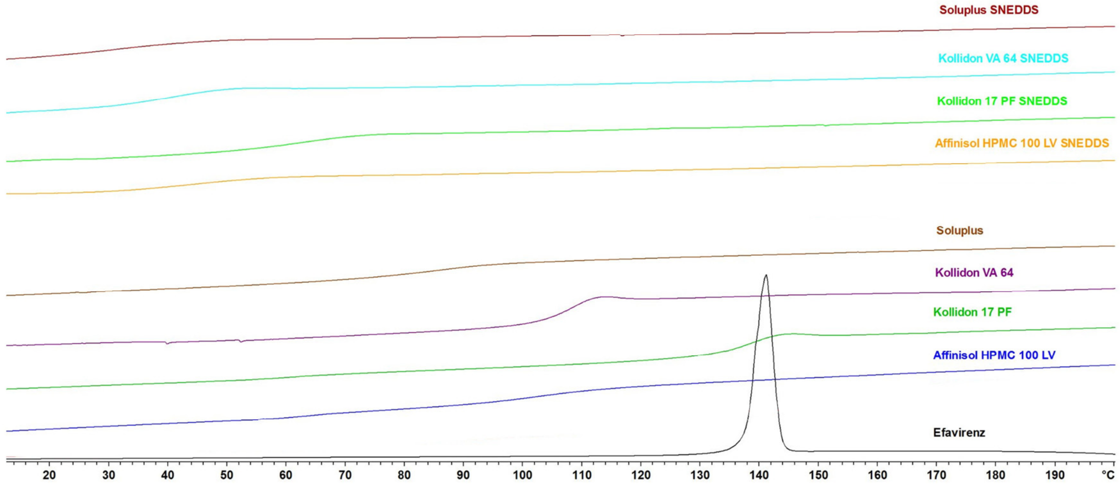

(b)

Figure S5. DSC thermograms (after six months of storage at 30 °C/65% RH) of efavirenz, ModE copolymers, and the corresponding S-SNEDDS (a), efavirenz and other marketed (co)polymers used in the study, and the corresponding S-SNEDDS (b).

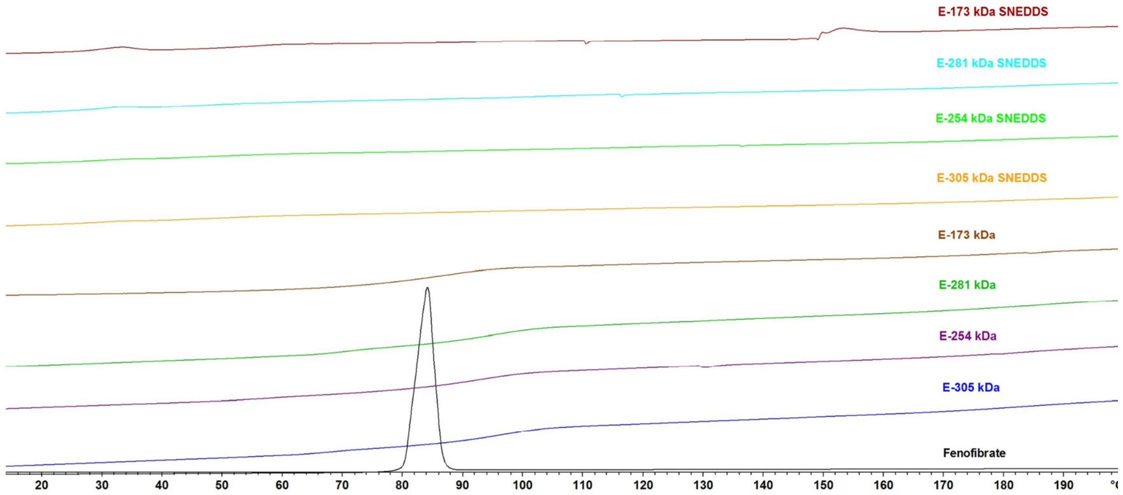

(a)

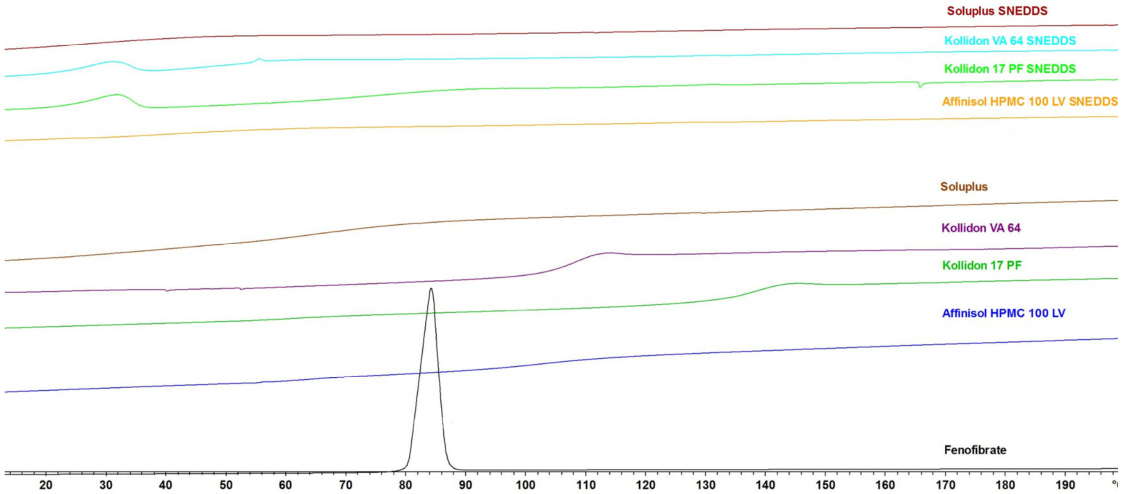

(b)

Figure S6. DSC thermograms (after six months of storage at 30 °C/65% RH) of fenofibrate, ModE copolymers, and the corresponding S-SNEDDS (a), fenofibrate and other marketed (co)polymers used in the study, and the corresponding S-SNEDDS (b).

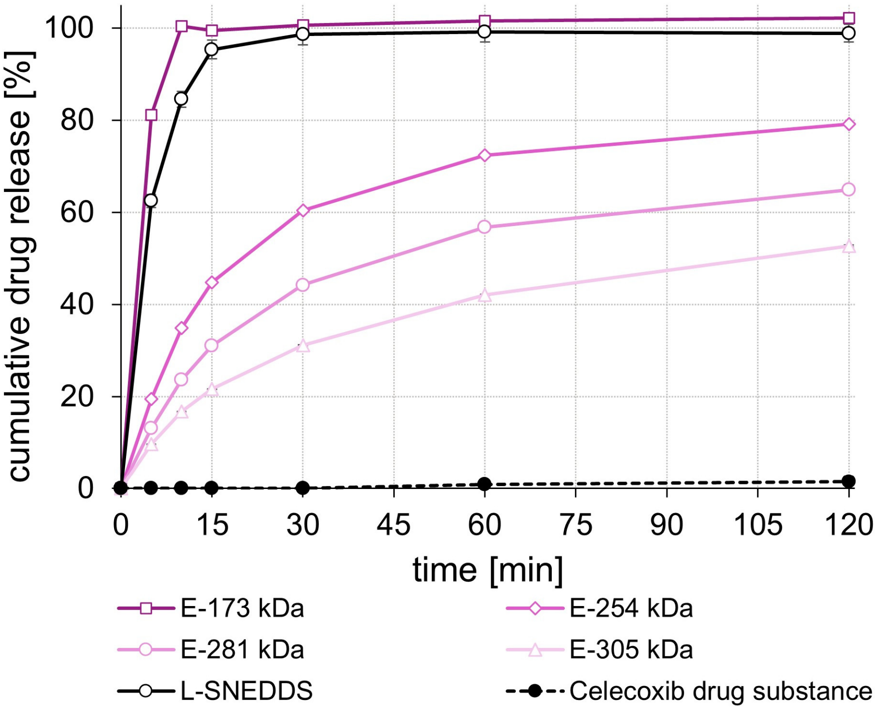

(a)

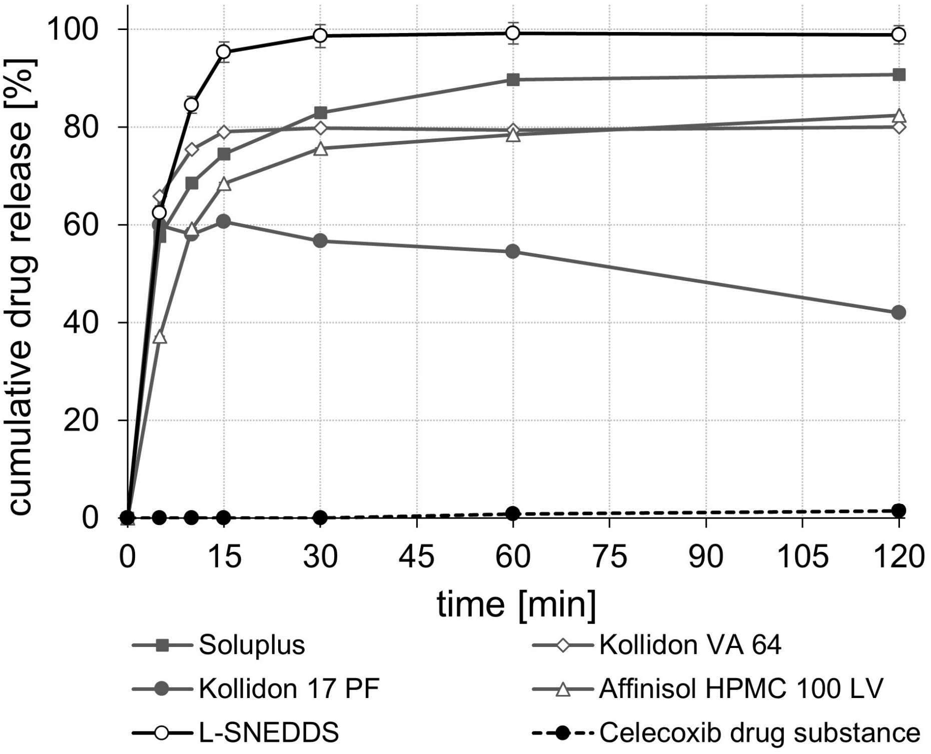

(b)

Figure S7. Dissolution profiles of celecoxib drug substance and celecoxib L- and S-SNEDDS based on ModE (a), as well as celecoxib drug substance and celecoxib L- and S-SNEDDS based on other marketed (co)polymers (b) (after three months of storage at 30 °C/65% RH) in 500 mL 0.1 M HCl in USP apparatus II. Each value designates the mean ± S.D. (*n* = 3).

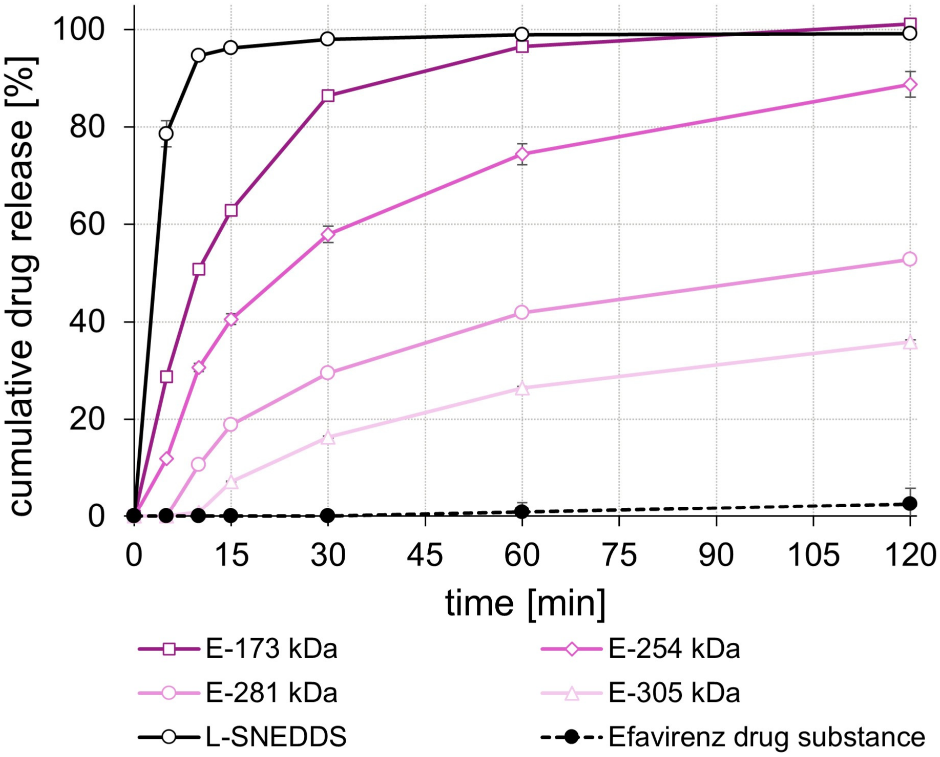

(a)

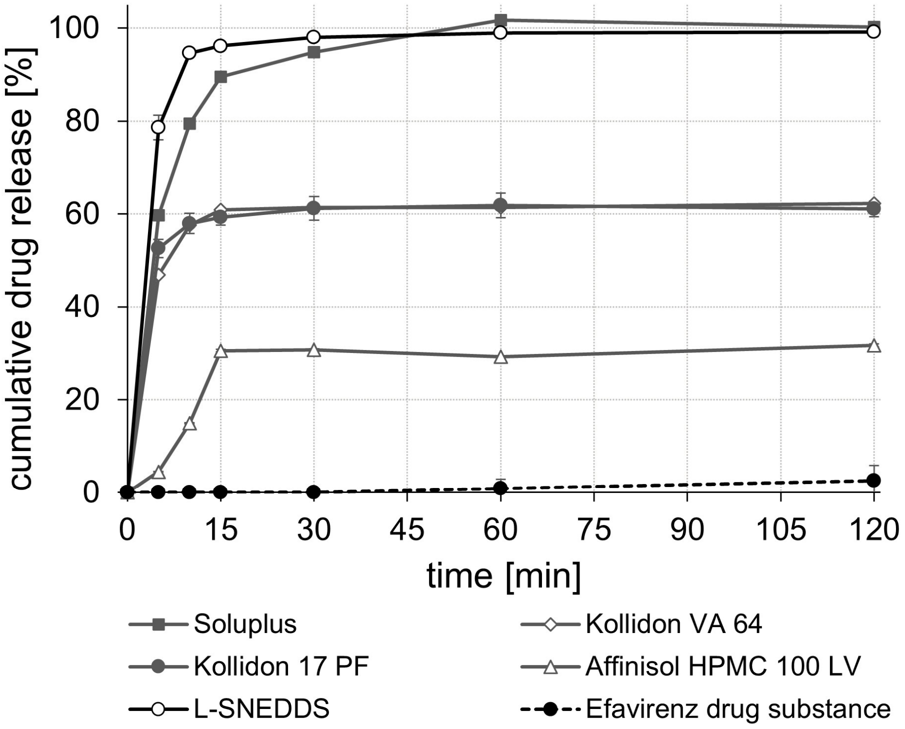

(b)

Figure S8. Dissolution profiles of efavirenz drug substance and efavirenz L- and S-SNEDDS based on ModE (a), as well as efavirenz drug substance and efavirenz L- and S-SNEDDS based on other marketed (co)polymers (b) (after three months of storage at 30 °C/65% RH) in 500 mL 0.1 M HCl in USP apparatus II. Each value designates the mean ± S.D. (n = 3).

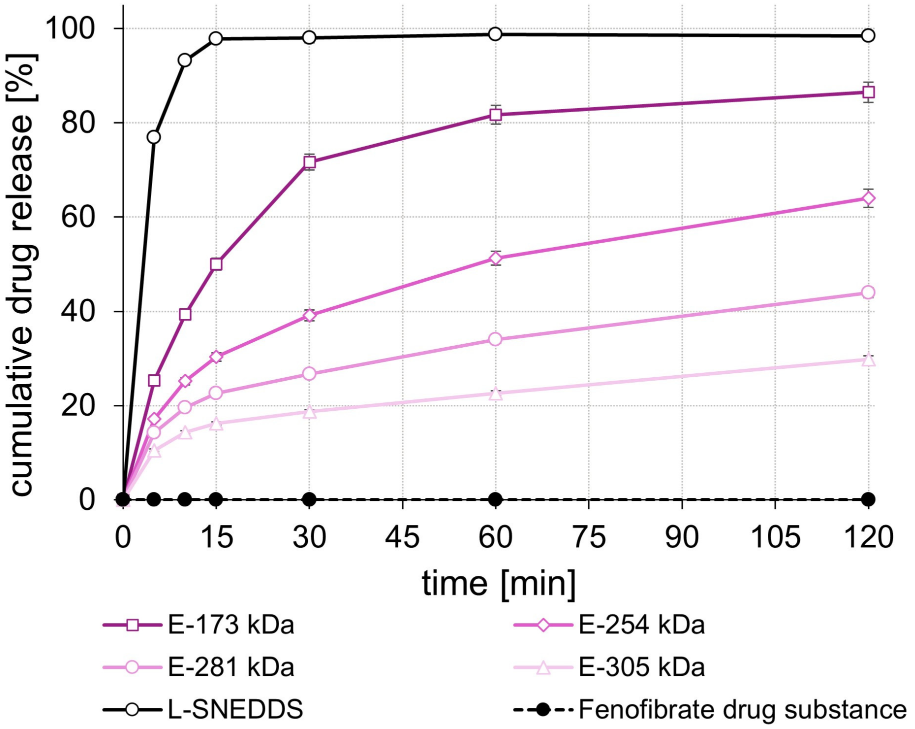

(a)

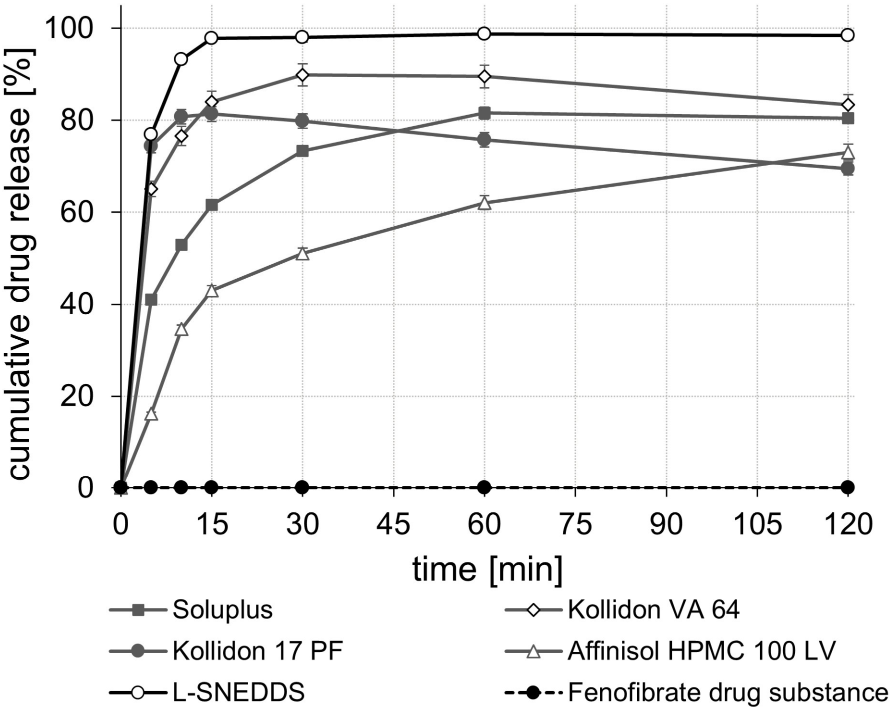

(b)

Figure S9. Dissolution profiles of fenofibrate drug substance and fenofibrate L- and S-SNEDDS based on ModE (a), as well as fenofibrate drug substance and fenofibrate L- and S-SNEDDS based on other marketed (co)polymers (b) (after three months of storage at 30 °C/65% RH) in 500 mL 0.1 M HCl in USP apparatus II. Each value designates the mean ± S.D. (n = 3).
